# Supplementary material for: Autoantibodies from Patients with Scleroderma Renal Crisis Promote PAR-1 Receptor Activation and IL-6 Production in Endothelial Cells
Source: Int J Mol Sci. 2021 Oct 30;22(21):11793. doi: 10.3390/ijms222111793 (PMC8584031; doi:10.3390/ijms222111793)
Supplement: Supplementary file 1 [file ijms-22-11793-s001.zip › ijms-1409640-supplementary.pdf]

International Journal of *Molecular Sciences*

MDPI

Supplementary Files

Autoantibodies from Patients with Scleroderma Renal Crisis Promote PAR-1 Receptor Activation and IL-6 Production in Endothelial Cells

Michèle Simon [1], Christian Lücht [1], Isa Hosp [1], Hongfan Zhao [1], Dashan Wu [1], Harald Heidecke [2], Janusz Witowski [1,3], Klemens Budde [1], Gabriela Riemekasten [4] and Rusan Catar [1,*]

[1] Department of Nephrology and Internal Intensive Care Medicine, Charité—Universitätsmedizin Berlin, Corporate Member of Freie Universität Berlin and Humboldt-Universität zu Berlin, 10117 Berlin, Germany; michele.simon@charite.de (M.S.); christian.luecht@charite.de (C.L.); isa@lutzschramm.de (I.H.); hongfan.zhao@charite.de (H.Z.); Dashan.wu@charite.de (D.W.); jwitow@ump.edu.pl (J.W.); klemens.budde@charite.de (K.B.)
[2] CellTrend GmbH, 14943 Luckenwalde, Germany; heidecke@celltrend.de
[3] Department of Pathophysiology, Poznan University of Medical Sciences, 60-806 Poznan, Poland
[4] Clinic for Rheumatology and Clinical Immunology, Universitätsklinikum Schleswig-Holstein, Lübeck, Germany; Gabriela.Riemekasten@uksh.de
* Correspondence: rusan.catar@charite.de; Tel.: +49-30-450-559248

Table S1. Demographic characteristics of the patients with SRC as well as anti-AT1-R, ETA-R and PAR-1 antibody levels detected.

	**Patient no.**	**1**	**2**	**3**	**4**	**median (min-max) frequency (%)**
**Patient characteristics**	**age [years]**	47	58	42	53	50.0 (42-58)
	**sex**	f	m	f	m	2/4 (50%) female
	**disease duration [month]**	91	68	82	25	80.5 (5-91)
	**diffuse/limited SSc**	limited	diffuse	diffuse	diffuse	3/4 (50%) diffuse
	**antibodies**	-	Scl-70	Scl-70	Scl-70	3/4 (75%) Scl-70 pos.
	**mean AT_1R-IgG level [U/mL]**	16.94	18.45	26.12	21.38	19.92 (16.94-26.12)
	**mean ET_AR-IgG level [U/mL]**	17.00	22.41	19.65	23.67	21.03 (17.00-23.67)
	**mean PAR-1-IgG level [U/mL]**	2.01	4.12	5.34	3.81	3.96 (2.01-5.34)
**Clinical presentation**	**hypertensive (≥140/85 mmHg)**	+	-	-	+	2/4 (50%)
	**systolic [mmHg]**	>300	105	180	130	155 (105->300)
	**diastolic [mmHg]**	140	80	110	95	102.5 (60-140)
	**initial kidney function/creatinine [mg/dL]**	3.64	4.02	4.82	3.71	4.00 (3.86-4.82)
**Histology**	**preglomerular TMA**	+	-	+	-	1/4 (50%)
	**glomerular TMA**	+	-	+	-	2/4 (50%)
	**fibrinoid necrosis**	+	-	+	-	2/4 (50%)
	**myxoid intimal deposition**	+	+	+	-	3/4 (75%)
	**concentric intimal sclerosis**	+	+	+	+	4/4 (100%)
	**IF/TA**	20%	15%	20%	20%	3/4 (75%)

Data are presented as frequency (%) or median (min-max).m-male; f - female; Scl-70 – anti-topoisomerase I; TMA - thrombotic microangiopathy; IF/TA - interstitial fibrosis/tubular atrophy .

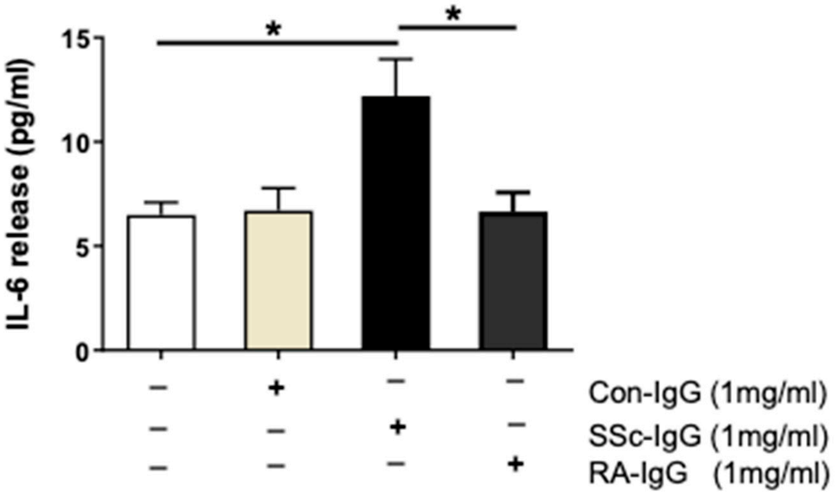

Figure S1. Effect of SSc-IgG and RA-IgG on IL-6 production by human microvascular endothelial cells. HMECs were treated with IgG from healthy controls (Con-IgG) or stimulated in the presence or absence of SSc-IgG and RA-IgG at the same dose (1 mg/ml) for 24 h (n=5). ANOVA mean +/− SEM with * $p < 0.05$.

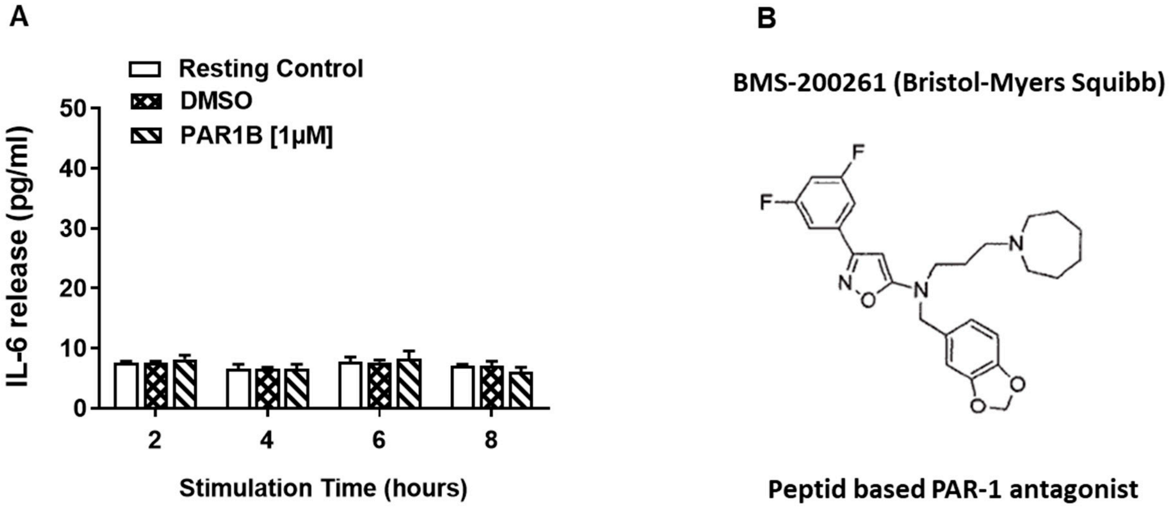

Figure S2. Effect of PAR-1 inhibition on IL-6 production. **(A)** HMECs were cultured in the presence of the solvent DMSO or BMS-200261 (peptid-based PAR-1 antagonist at 1 µM) for 60 min and assessed for time-dependent IL-6 secretion (n=4). ANOVA means ± SEM with * $p < 0.05$. **(B)** Structural chemistry of the peptid based PAR1 antagonist BMS-200261.

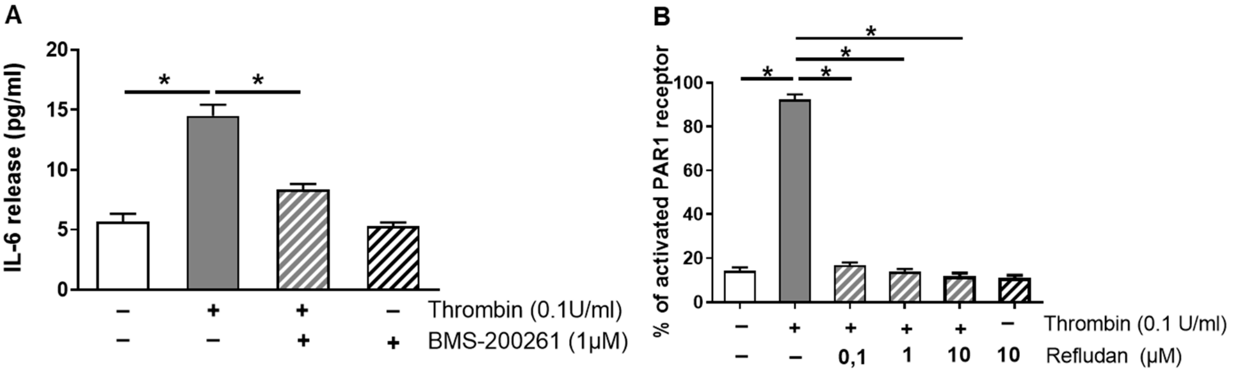

Figure S3. Effect of thrombin on IL-6 production and PAR-1 activation. HMECs were stimulated with thrombin (0.1 U/mL) for 60 min and assessed for **(A)** IL-6 secretion or **(B)** PAR-1 activation by measuring the percentage of cleaved PAR-1 on the cell surface. To verify the specificity of the effect of thrombin, HMECs were pretreated for 1 h with **(A)** either BMS-200261 (PAR-1 antagonist) or **(B)** the thrombin inhibitor Lepirudin/Refludan. The data are presented as ANOVA means ± SEM from 4 experiments with * $p < 0.05$.
